# Relationships between Mucosal Antibodies, Non-Typeable *Haemophilus influenzae* (NTHi) Infection and Airway Inflammation in COPD

**DOI:** 10.1371/journal.pone.0167250

**Published:** 2016-11-29

**Authors:** Karl J. Staples, Stephen Taylor, Steve Thomas, Stephanie Leung, Karen Cox, Thierry G. Pascal, Kristoffer Ostridge, Lindsay Welch, Andrew C. Tuck, Stuart C. Clarke, Andrew Gorringe, Tom M. A. Wilkinson

**Affiliations:** 1 Clinical & Experimental Sciences, University of Southampton Faculty of Medicine, Southampton General Hospital, Tremona Road, Southampton, United Kingdom; 2 Wessex Investigational Sciences Hub, University of Southampton Faculty of Medicine, Southampton General Hospital, Tremona Road, Southampton, United Kingdom; 3 Public Health England, Porton Down, Salisbury, United Kingdom; 4 GSK Vaccines, Wavre, Belgium; 5 Southampton NIHR Respiratory Biomedical Research Unit, Southampton General Hospital, Tremona Road, Southampton, United Kingdom; Imperial College London, UNITED KINGDOM

## Abstract

Non-typeable *Haemophilus influenzae* (NTHi) is a key pathogen in COPD, being associated with airway inflammation and risk of exacerbation. Why some patients are susceptible to colonisation is not understood. We hypothesised that this susceptibility may be due to a deficiency in mucosal humoral immunity. The aim of our study (NCT01701869) was to quantify the amount and specificity of antibodies against NTHi in the lungs and the associated risk of infection and inflammation in health and COPD. Phlebotomy, sputum induction and bronchoscopy were performed on 24 mild-to-moderate COPD patients and 8 age and smoking-matched controls. BAL (Bronchoalveolar lavage) total IgG1, IgG2, IgG3, IgM and IgA concentrations were significantly increased in COPD patients compared to controls. NTHi was detected in the lungs of 7 of the COPD patients (NTHi+ve—29%) and these patients had a higher median number of previous exacerbations than NTHi-ve patients as well as evidence of increased systemic inflammation. When comparing NTHi+ve versus NTHi-ve patients we observed a decrease in the amount of both total IgG1 (p = 0.0068) and NTHi-specific IgG1 (p = 0.0433) in the BAL of NTHi+ve patients, but no differences in total IgA or IgM. We observed no evidence of decreased IgG1 in the serum of NTHi+ve patients, suggesting this phenomenon is restricted to the airway. Furthermore, the NTHi+ve patients had significantly greater levels of IL-1β (p = 0.0003), in BAL than NTHi-ve COPD patients.This study indicates that the presence of NTHi is associated with reduced levels and function of IgG1 in the airway of NTHi-colonised COPD patients. This decrease in total and NTHI-specific IgG1 was associated with greater systemic and airway inflammation and a history of more frequent exacerbations and may explain the susceptibility of some COPD patients to the impacts of NTHi.

## Introduction

Non-typeable *Haemophilus influenzae* (NTHi) is a non-capsulated bacterium that can cause respiratory tract infections throughout life[1]. NTHi can also chronically infect the airways of patients with COPD and is associated with greater airway inflammation[2–4] and with acute exacerbations[2, 5]. Microbiological studies of the COPD airway have identified that the presence of NTHi is associated with loss of microbial diversity[6] and that dynamic changes in NTHi strain carriage contribute to risk of exacerbation[5]. Whilst there is growing evidence from a number of sources for an impaired immune response in the COPD lung[7–9] the mechanisms which lead to susceptibility to NTHi infection are not yet understood.

When comparing COPD patients to healthy controls, total serum immunoglobulin (Ig) levels are not reduced, suggesting that it is not a deficiency in overall systemic humoral immunity that underlies risk of airway infection[10]. In fact, the evidence points to certain antibodies (IgD) being elevated in the serum of COPD patients[10]. Previous work has found NTHi infection of the COPD lung despite detection of specific IgG and IgA antibodies in both serum and sputum[11]. However, a decrease in the amount of secretory (s)IgA in the bronchoalveolar lavage (BAL) of COPD patients, has been recently reported in advanced disease[7]. In contrast there have also been reports of increased IgA in COPD lung tissue[12], but to date no study has comprehensively analysed immunoglobulin levels in the human airway in the context of NTHi infection, or in earlier stages of disease. Indeed the immune correlates of protection against this un-encapsulated organism are not fully understood, but murine models of COPD have demonstrated impaired immunoglobulin class switching in disease with associated effects on NTHi clearance[13]. Whether this is a relevant mechanism in man is not understood.

The aim of this study was to explore the differences in airway immunoglobulin levels and associated functional competence of mucosal antibody-mediated immunity to NTHi in health and COPD. We further wished to assess how these aspects of mucosal immunity were related to airway infection and inflammation in stable disease.

## Materials and Methods

### Ethics

All subjects gave written informed consent and the study (ClinicalTrials.gov: NCT01701869) was approved by the National Research Ethics Service (NRES) Southampton B Committee (12/SC/0304).

### Subjects

**Fig 1:**
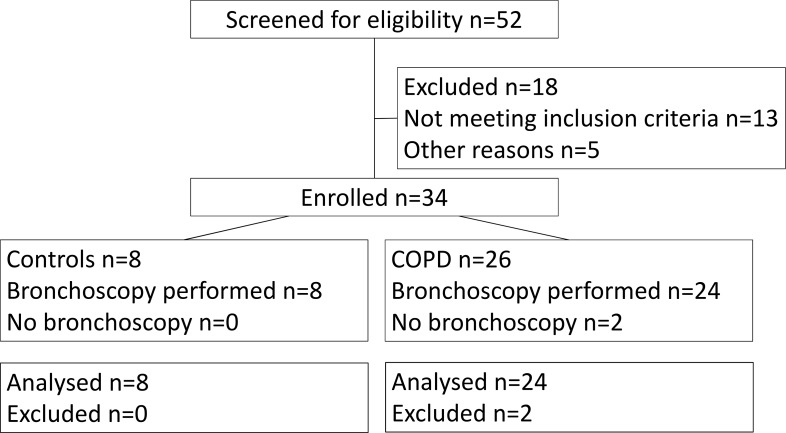
Volunteer recruitment CONSORT diagram. Subjects who did not undergo bronchoscopy were excluded based on HRCT finding of other lung pathologies.

Twenty-four subjects with stable mild and moderate COPD [14] were recruited. COPD diagnosis was confirmed by post-bronchodilator spirometry with a FEV1/FVC ratio of <0.7 and an FEV1 of ≥50% predicted value required for enrolment and bronchoscopy. Spirometry was conducted in accordance with ATS standards[15]. Subjects had at least a smoking history of 10 pack years. Exclusion criteria included a history of other pulmonary disease, alpha-1-antitrypsin deficiency, long-term antibiotics or oral corticosteroids or an exacerbation within the month prior to recruitment. A control group of eight current or ex-smokers, with at least a 10 pack year history but preserved lung function were also recruited. CONSORT diagram is reproduced in the online supplement (Fig 1).

### Lung sample acquisition

Sputum was obtained by the inhalation of nebulised hypertonic saline as previously described[16]. Fibreoptic bronchoscopy was performed on an outpatient basis. In each subject two lobes were targeted and two protected brushes per lobe were used to sample the airway microbiome. BAL was performed by separately instilling 100 ml of 0.9% (w/v) saline into each lobe and each aliquot was recovered by aspiration. The separate aliquots of BAL fluid were then poured through 100 μm filters and cells removed by 400 g centrifugation for 10 min at 4⁰C. The supernatant was aliquoted and samples from different lobes stored separately at -80⁰C prior to analysis. The resulting cell pellets were prepared separately for cytospin analysis as previously described[17] and flow cytometry analysis.

### Microbiological analysis

Culture of nasal brushes, protected bronchial brushes and sputum was performed. Briefly, an equal volume of 0.1% dithiothreitol (sputolysin) was added to 100 μl of sputum prior to plating. Brush samples were collected in 5 ml PBS, which was then centrifuged at 400 g, 4°C for 5 min to concentrate the cellular sample. Pellets were then resuspended in 250 μl PBS prior to plating. *H*. *influenzae* was identified by colony morphology on chocolate agar (Oxoid, Basingstoke, UK) and requirement of X+V growth factors (Oxoid, Basingstoke, UK) on blood base agar (Oxoid, Basingstoke, UK) after an overnight incubation period at 37°C in 5% CO_2_. Positive isolates were inoculated into STGG (BioTrading, Mijdrecht, The Netherlands) and stored at -70°C.

For PCR detection of *H*. *influenzae*, DNA was extracted from DTT-treated induced sputum or PBS-containing brush samples using the MagNA Pure 96 DNA and Viral NA Small Volume Kit (Roche Diagnostics), as per the manufacturer’s instructions. A quantitative polymerase chain reaction (PCR) targeting *lgtC* gene was used for the detection of *H*. *influenzae*. Quantitative PCR for NTHi detection was then performed. The limit of detection of this assay was 2000 copies/ml, which was used as positivity cut-off.

Isolates were removed from frozen storage and inoculated onto chocolate agar (Oxoid, Basingstoke, UK) for overnight incubation at 37°C in 5% CO2. Colonies were suspended in 180μl of ATL Buffer (QiaGen, Manchester, UK). To each suspension 20μl of proteinase K (QiaGen, Manchester, UK) was added and heated at 56°C for two hours in a waterbath. DNA extraction was performed to the manufacturer’s instructions using the QiaAmp mini kit (QiaGen, Manchester, UK). DNA quantification was carried out using a Qubit flurometer (Life Technologies, Paisley, UK) according to manufacturer’s instructions and samples normalised to 0.2ng μl-1 by dilution in DNAse/RNAse free water (Gibco, Paisley,UK). The Nextara XT DNA prep kit was used for library preparation of the isolates (Illumina, Saffron Walden, UK) and Illumina MiSeq V2 2 x 250bp paired end sequencing performed to manufacturer’s instructions. All short read sequence files achieved quality score of >80% at Q30 and were therefore deemed successful.

### Multi Locus Sequence Typing

The multi locus sequence typing (MLST) schema for *H*. *influenzae* was downloaded from SRST2[18, 19]. SRST2 was used to perform *in-silico* MLST analysis on all isolates, all resulting in coverage >30.

### In-silico PCR for identification of NTHi *iga* gene

Previously published primers were used in iPCRess for *in-silico* PCR analysis to ascertain presence of *iga* responsible for iga protease translation in NTHi[20]. Forward primer *iga*BF1 TGAATAACGAGGGGCAATATAAC and reverse primer *iga*BR1 TCACCGCACTTAATCACTGAAT [21]. The mismatch setting in iPCRess was set to 3.

### Mapping to reference sequences for the NTHi *iga* and *igaB* protease genes

Reference sequences for the conserved beta core sequence of *iga* and full CDS of *iga*B, genes responsible for iga protease production in *H*. *influenzae* were obtained from GenBank (iga–GenBank accession no M87492, bases 4124–4978 *iga*B GenBank accession number KC607498.1). Isolates were mapped against these reference sequences using SRST2 with settings of minimum coverage at 60 and invoking the ‘report_all_consensus_alleles’ option [19].

### Serum and BAL supernatant analyses

Total immunoglobulin isotype concentrations were quantified using the MSD platform according to the manufacturer’s instructions (MSD, Rockville, USA). Secretory IgA was quantified using ELISA according to the manufacturer’s instructions (Demeditec Diagnostics GmbH, Kiel, Germany). Cytokine and MMP concentrations in BAL were quantified using a Luminex multiplex immunoassay (R&D systems, Abingdon, UK). Samples were analysed on the Luminex 200 platform (Biorad Bioplex 200, Hemel Hempstead, UK), as per manufacturer’s instructions. Cytokine analysis was performed for IL-1β, IL-2, IL-6, IL-8, IL-10, GM-CSF, IFNγ and TNFα. Matrix metalloprotease (MMP) analysis was performed for MMP-1, -2, -3, -7, -8, -9, -10, -12, -13 and the ECM metalloprotease inducer (EMMPRIN).

### Assessment of NTHi-specific antibodies by immunoglobulin binding assay (IBA)

Binding of antibodies to NTHi was determined by flow cytometry. NTHi 3224A (sequence type (ST) 259—obtained from GSK Vaccines) was plated onto chocolate agar, supplemented with PolyVitex (Biomerieux SA, France), and incubated at 37°C with 5% CO_2_ for 16–24 h. Bacteria were resuspended in 2.5 ml brain heart infusion broth (Oxoid) supplemented with haemin (Sigma) and nicotinamide adenine dinucleotide (Sigma). This was incubated with shaking for 2–3h at 37°C until the OD620nm was 0.35–0.45. Two microliters of heat inactivated sera (56°C for 30 min) was added to appropriate wells of a standard U-bottom 96-well microtitre plate. 198 μl of bacteria, at OD620nm 0.1, in blocking buffer (IBA-BB) (2% BSA in 1x PBS w/v) was added to every well and the plate incubated at 25°C for 30 min with shaking at 900 rpm. The plate was centrifuged at 3060g for 5 min, each well washed with 200 μl IBA-BB, centrifuged again and the pellets re-suspended in 200μl fluorescent detection antibody conjugated with fluorescein isothiocyanate (FITC) or 200 μl unconjugated secondary antibody, diluted 1:500 in IBA-BB. The conjugated detection antibodies used were either goat anti-human FITC or goat anti-mouse FITC, depending on whether the assay used test sera (human) or test sera and a secondary antibody (mouse). Where the assay was detecting just the test antibody the plate was incubated for 20 min at 4°C then centrifuged at 3060 *g* for 5 min, washed twice with 200 μl IBA-BB, and finally centrifuged again and the pellets re-suspended in 200 μl IBA-BB. Where the assay was detecting a secondary antibody the plate was incubated for a further 20 min at 4°C, after the first of the two wash steps mentioned previously, before being washed twice with IBA-BB. The plate was stored in the dark at 4°C until analysed by flow cytometry. All tests were performed in duplicate and the following background controls were used in the assay: bacteria only and bacteria plus conjugate only.

### Oxidative burst assay (OBA)

Bacteria were prepared at 5.0x10^9^/ml in blocking buffer (OBA-BB) (2% Marvel in HBSS+Ca2++Mg2+). 5 μl heat inactivated sera were added to appropriate wells of a standard U-bottom 96-well plate. 15 μl of OBA-BB was added to wells containing sera, or an appropriate amount to control wells to give final volume of 40 μl prior to addition of HL60 cells. 10 μl bacteria was added to every well except “cells only” control. The plate was incubated for 15 min at 37°C with shaking at 900 rpm. 10 μl IgG-depleted human plasma (diluted 1:10 in OBA-BB) was added to appropriate wells. This was then incubated for 7.5min at 37°C with shaking at 900 rpm. Differentiated HL60 cells at 2.5x10^7^/ml were prepared by centrifuging at 400 *g* for 5min and then re-suspending in OBA-BB. 25 μl prepared cells were added to all wells along with 25 μl 25 μg/ml Dihydrorhodamine 123 (DHR 123 –Life Technologies, D23806), and incubated for 15 min at 37°C with shaking at 900 rpm. Microtitre plates were immediately placed on ice and assay fixed with 80 μl 1% formaldehyde in DPBS+0.02% (w/v) EDTA and incubated for 30 min at RT covered with foil, to eliminate light. Wells were analysed immediately on the flow cytometer.

### Bacterial flow cytometry

Assays were analysed using a Beckman Coulter Cyan flow cytometer equipped with a Cytek 96-well microtitre plate reader. Protocols were initially set-up to analyse profiles of events identified on the cytometer by the forward scatter (FS), measuring the size of the cell, and side scatter (SS), measuring the granularity and internal structural complexity. An analysis ‘gate’ was drawn around the population of interest and a relevant histogram plot created to analyse the fluorescence given off by the event population. For each sample, 10,000 individual events were analysed for fluorescence and a horizontal gate was drawn to include ~10% of the control sample population (bacteria plus conjugate for IBA and cells plus bacteria plus complement for OBA). A Fluorescence Index (FI) was calculated for each sample, which involved the multiplication of the % of events moving into the horizontal gate (%-gated), by the average fluorescence of that population (X-mean). The final result for each test was expressed as the average FI (average FI taken for duplicate test samples) of the test serum sample minus the average FI of the bacteria plus conjugate only control for IBA, expressed as FI-Conj and cells plus bacteria plus complement control for OBA, expressed as FI-C’.

### Statistics

Analysis of three groups was performed using a Kruskal-Wallis ANOVA followed by a Dunn’s *post hoc* test. Analysis of two groups was performed using a one-tailed Man-Whitney U test for analysis of previous exacerbations and NTHi-specific Ig when a previous Kruskal-Wallis test had already indicated a difference in total Ig levels. Fishers exact test was used for categorical data (GraphPad Prism v6, GraphPad Software Inc., San Diego, USA). Each subject had two lobes sampled and the mean concentrations of the separate aliquots from each lobe were used. Associations between Ig and spirometry parameters were assessed using Spearman’s correlation with rho and p values presented. Results were considered significant if p<0.05.

## Results

### Volunteers

The clinical characteristics of volunteers are presented in Table 1. COPD patients were matched with controls for age, but had significantly lower FEV_1_% predicted and greater airflow obstruction. The proportion of BAL macrophages from COPD patients was lower with a corresponding increase in the proportion of neutrophils highlighting the increased inflammation in the COPD airway.

**Table 1:** Clinical characteristics of volunteers.

	Controls	NTHI-ve	NTHi+ve
N	8	17	7
Age (years)	56 (47.75–65.75)	66 (57.00–69.50)	68 (60.00–72.00)
Gender M/F	6 / 2	13 / 4	3 / 4
BMI	26.06 (24.25–29.83)	29.03 (26.05–31.84)	28.13 (27.60–30.36)
Current Smoker	5	6	5
Pack Years	32.25 (21.25–45.00) (Range 10–55)	38.00 (27.50–64.00) (Range 15–160)	50.00 (12.00–80.00) (Range 10–90)
FEV_1_, % predicted	108 (101–121.3)	71.00** (61.00–80.00)	62.00*** (52.00–76.00)
FEV_1_/FVC ratio	0.78 (0.73–0.82)	0.56*** (0.53–0.63)	0.51*** (0.46–0.61)
Inhaled corticosteroids, Y/N	N/A	11/6	5/2
Daily steroid dose, μg (BDP equivalent)	N/A	1000 (800–2000)	1000 (800–2000)
Exacerbations in previous year, N	N/A	1.0 (1.0–2.0)	3.0# (1.0–6.0)
Blood WCC, 10^9^/L	7.95 (6.83–8.98)	6.50 (5.40–7.30)	8.30† (7.50–9.80)
Blood neutrophils, 10^9^/L	4.5 (3.85–5.20)	3.70 (3.10–4.40)	4.90 (3.90–5.30)
Blood lymphocytes, 10^9^/L	2.35 (1.85–2.75)	1.90 (1.80–2.10)	2.60† (2.10–3.30)
BAL macrophage, %	97.25 (88.38–98.25)	81.75* (46.25–89.50)	90.00 (69.50–93.50)
BAL neutrophil, %	0.75 (0.13–1.00)	5.25* (2.00–8.75)	2.00 (1.50–16.00)
BAL lymphocyte, %	0.25 (0.00–0.875)	0.00 (0.00–2.50)	1.00 (0.50–2.50)

Data are presented as median and IQR. For pack years, the minimum and maximum values are also presented. Ex-smokers were defined as individuals who had stopped smoking for > 6 months. Blood data shown represents 8 controls, 15 NTHi-ve and 7 NTHi+ve COPD volunteers. BAL data shown represents 8 controls and 14 NTHI-ve and 5 NTHI+ve COPD volunteers. Data were analysed using a Kruskal-Wallis ANOVA with Dunn’s post hoc comparison.

* p<0.05

** p<0.01

*** p<0.001 vs Controls

† p<0.05 vs NTHI-ve.

# p<0.05 One-tailed Mann Whitney U Test.

### IgA is increased in the COPD airway

**Fig 2:**
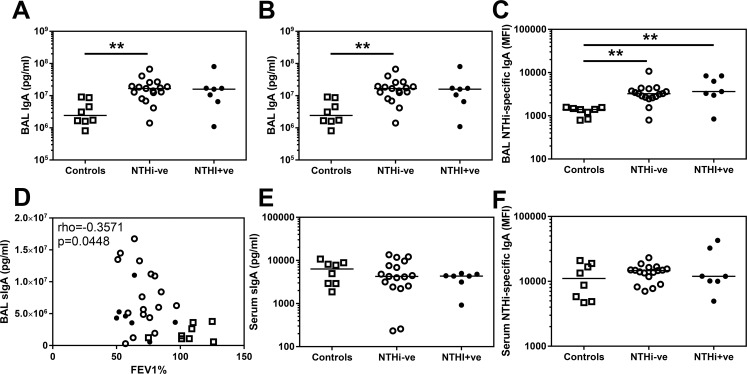
IgA and sIgA levels in BAL and serum derived from controls and COPD patients. **(A)** Total IgA and **(B)** secretory IgA in BAL derived from two lung lobes were analysed by MSD multiplex assay and ELISA respectively and the average concentration from both lobes is presented. **(C)** Assessment of BAL IgA specific for the control strain of NTHi (3224A) were assessed by flow cytometry. **(D)** Secretory IgA in BAL derived from all volunteers are plotted against FEV1% and analysed using a Spearman’s correlation with rho and p value presented. Assessment of **(E)** secretory IgA in serum measured by ELISA and **(F)** serum IgA specific for the control strain of NTHi (3224A) were assessed by flow cytometry. Open squares indicate controls, open circles indicate NTHi-ve COPD patients, closed circles indicate NTHi+ve COPD patients. Bars represent median values and each dot represents an individual volunteer n = 8 for controls and n = 17 for NTHi-ve COPD patients and n = 7 for NTHi+ve COPD patients. Data were analysed using a Kruskal-Wallis ANOVA followed by a Dunn’s post hoc test ** p<0.01.

There are conflicting previous reports of both decreased sIgA[7] and increased IgA[12] in the COPD airway. We therefore assessed both total and secretory IgA in BAL (Fig 2A and 2B). We observed significantly greater amounts of both these isoforms in COPD BAL compared to controls. Moreover, there was also significantly greater amounts of NTHi-specific IgA in BAL from COPD patients (Fig 2C). In contrast to a previous report demonstrating a positive correlation between sIgA in BAL and lung function[7], we observed a significant inverse correlation with higher amounts of sIgA produced in those volunteers with worse FEV1% predicted (Fig 2D).

This increase in sIgA appeared to be limited to the airway as there was no significant difference in the amount of sIgA in the serum of controls (median 6340 pg/ml) and COPD patients (median 4335 pg/ml, Fig 2E). Correspondingly there was also no significant difference in the amount of NTHi-specific IgA in serum (Fig 2F).

### Immunoglobulin isotypes are increased in the COPD airway

**Fig 3:**
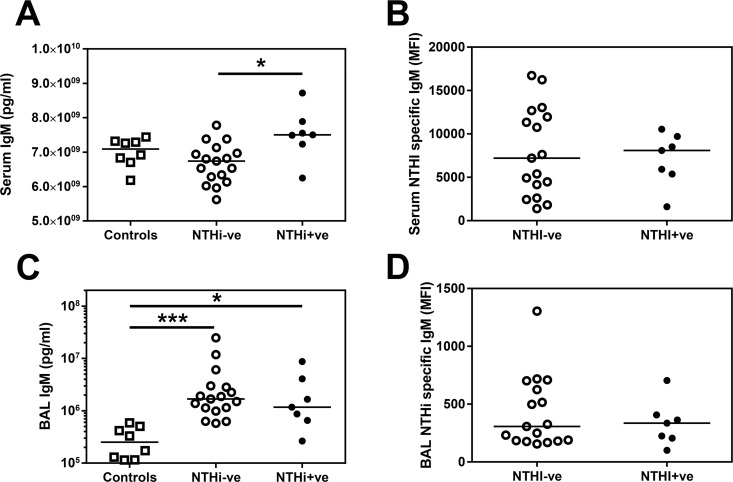
Total IgM but not NTHi-specific IgM are increased in the serum of NTHi+ve patients. Total IgM in **(A)** serum or **(C)** BAL was analysed by MSD multiplex assay. **(B)** Serum IgM **or (D)** BAL IgM specific for NTHi was assessed by flow cytometry. Open circles indicate NTHi-ve COPD patients, closed circles indicate NTHi+ve patients. Bars represent median values and each dot represents an individual volunteer. n = 8 for controls, n = 17 for NTHi-ve and n = 7 for NTHi+ve patients. Data were analysed using a Kruskal-Wallis ANOVA followed by a Dunn’s post hoc test. * p<0.05, *** p<0.01

**Fig 4:**
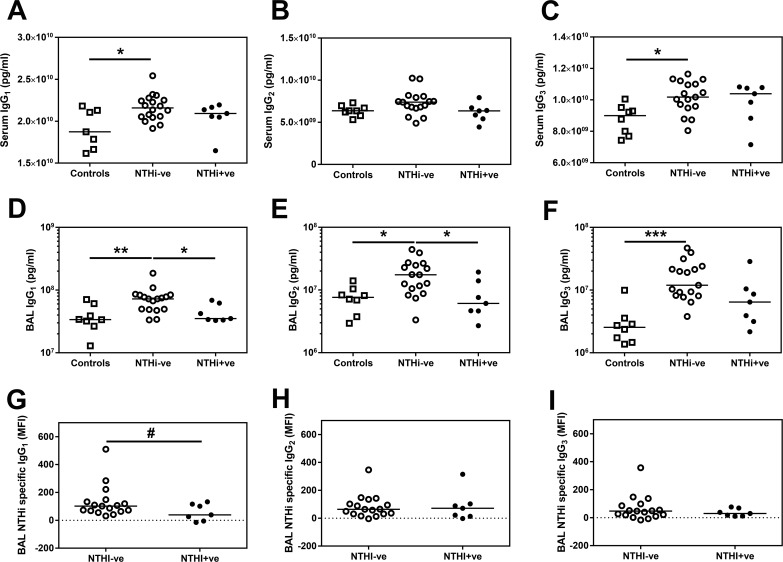
Total and NTHi-specific IgG1 are decreased in BAL from NTHi+ve patients. Total **(A)** serum IgG1, **(B)** serum IgG2 **(C)** serum IgG3 was analysed by MSD multiplex assay. Total **(D)** IgG1, **(E)** IgG2 and **(F)** IgG3 in BAL derived from two lung lobes were analysed by MSD multiplex assay and the average concentration from both lobes is presented. Bars represent median values and each dot represents an individual volunteer n = 8 for controls, n = 17 for NTHi-ve and n = 7 for NTHi+ve patients. Data were analysed using a Kruskal-Wallis ANOVA followed by a Dunn’s post hoc test. * p<0.05, ** p<0.01, *** p<0.01. **(G)** BAL IgG1, **(H)** BAL IgG2 or **(I)** BAL IgG3 specific for NTHI was assessed by flow cytometry. Open squares indicate controls, open circles indicate NTHi-ve COPD patients, closed circles indicate NTHi+ve patients. Bars represent median values and each dot represents an individual volunteer n = 17 for NTHi-ve and n = 7 for NTHi+ve patients. # p<0.05 using a one tailed Mann Whitney test.

To investigate whether this increase in both total and secretory IgA was specific to this antibody isotype or was applicable to all Ig in the COPD airway, we also quantified the amount of IgG and IgM in the BAL (Figs 3 & 4). Total IgM was significantly increased in the COPD airway compared to healthy airways (Fig 3C), as were total IgG1, IgG2 and IgG3 (Fig 4D–4F), but not IgG4 (See S1 Fig). This increase in the IgG subtypes and IgM in the COPD airway was also reflected by a trend towards a greater amount of NTHi-specific total IgG (Kruskal-Wallis p = 0.0484 –See S1 Fig) and IgM (Fig 3C) in COPD BAL.

**Fig 5:**
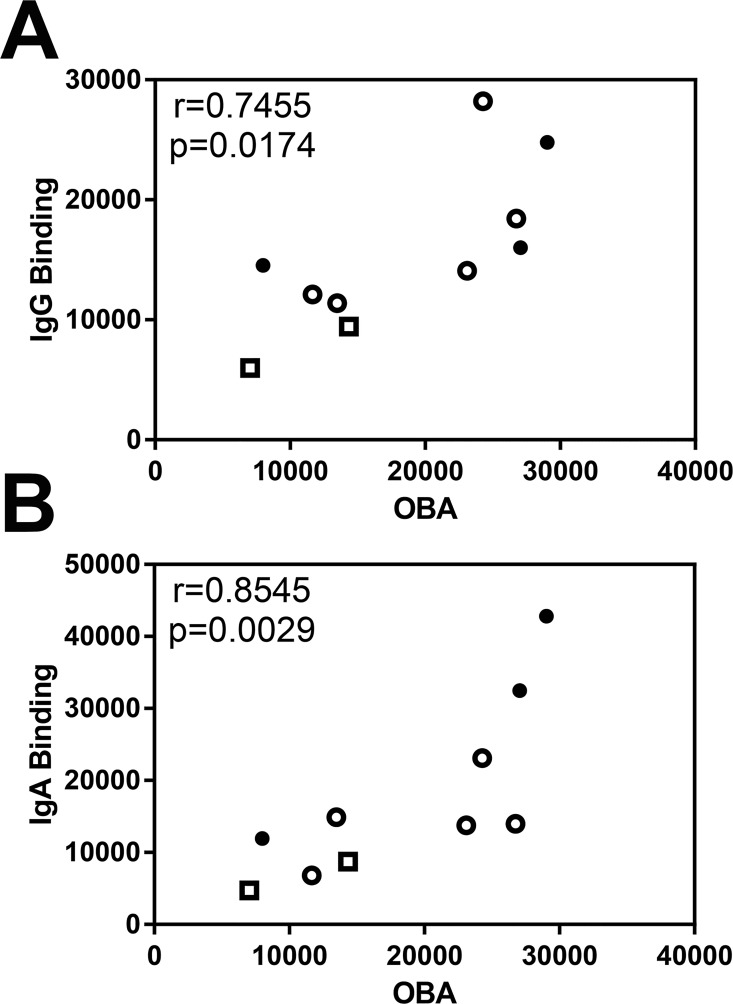
Correlation of NTHi-specific antibody binding with oxidative burst assay. Spearman’s correlation of NTHi-specific **(A)** total IgG binding with IgG specific oxidative burst assay (OBA) and **(B)** total IgA binding with IgA specific OBA in serum with rho and p value presented. Open squares indicate controls (n = 2), open circles indicate NTHi-ve COPD patients (n = 5), closed circles indicate NTHi+ve patients (n = 3).

In preliminary experiments comparing NTHi-specific antibody binding to Ig-dependent uptake of NTHi and oxidative burst in HL-60 cells, NTHi-induced oxidative burst correlated well with both NTHI-specific IgG (r = 0.7455) and IgA (r = 0.8545) binding (Fig 5A & 5B). These data suggest that specific antibody binding can be used as a surrogate marker for antibody-mediated phagocytosis of NTHi and subsequent oxidative burst.

In contrast to the IgA data, there were significantly greater amounts of IgG1 and IgG3 in the serum of COPD patients compared to controls (Fig 4A & 4C), but intriguingly there was no increase in serum IgM (Fig 3B). Given this evidence of increased specific immunoglobulins in both the airway and the serum, the question remains why some COPD patients are infected with NTHi despite the increased presence of protective antibodies in the airway?

### Antibody levels in BAL of NTHi infected patients

In order to perform a comparison of immunoglobulin levels associated with NTHi infection, we conducted a preplanned analysis to compare COPD patients who were infected with NTHi and those who were not. Of the 24 COPD patients recruited, seven were NTHi infected (5 on sputum or bronchial brush culture and a further 2 sputum PCR positive for the *lgtC* gene). We therefore compared the 7 patients in which NTHi was detected (NTHi+ve), with the remainder of the cohort who were NTHi-ve (n = 17). Analysis of the cultured bacteria revealed that each individual patient was only infected by one ST of NTHi (See Table A in S1 File).

There were no discernible differences in demographics or clinical characteristics between the two COPD groups apart from significantly greater incidence of previous exacerbations in the NTHi+ve patients suggesting a persistent clinical phenotype (Table 1). Furthermore, NTHi+ve patients had evidence of greater systemic inflammation with increased white cell count (WCC), which may largely reflect increased lymphocyte numbers (Table 1).

Total IgG1 levels were significantly reduced in BAL from NTHi+ve subjects compared to NTHi-ve subjects (Fig 4D). In the same samples, the amount of NTHi-specific IgG1 was also lower in NTHi+ve compared to NTHI-ve patients’ BAL (Fig 4G). This decrease appears specific to IgG1 as although there is a significantly reduced amount of total IgG2 in BAL from NTHi+ve patients compared to NTHI-ve patients (Fig 4E), there is no significant difference in the amount of NTHi-specific IgG2 (Fig 4H). There were also no significant differences in the total levels of IgG3 or NTHi-specific IgG3 in BAL (Fig 4F & 4I). Furthermore, the relationship with low IgG1 persisted even when patients were categorised based on NTHi culture alone (See Table B in S1 File). NTHi+ve patients had similar levels of IgG1 in BAL as HC volunteers (Fig 4G). Contrastingly, both IgA (Fig 2A–2C) and IgM (Fig 3C) remained elevated in NTHI+ve BAL compared to controls.

Total IgM levels were significantly higher in NTHi+ve patients’ serum which might suggest a systemic expression of the immune phenotype (Fig 3A). However, this did not correspond to a significant increase in NTHi-specific IgM in the serum (Fig 3B).

### Inflammatory milieu is altered in the NTHi-colonised airway

**Fig 6:**
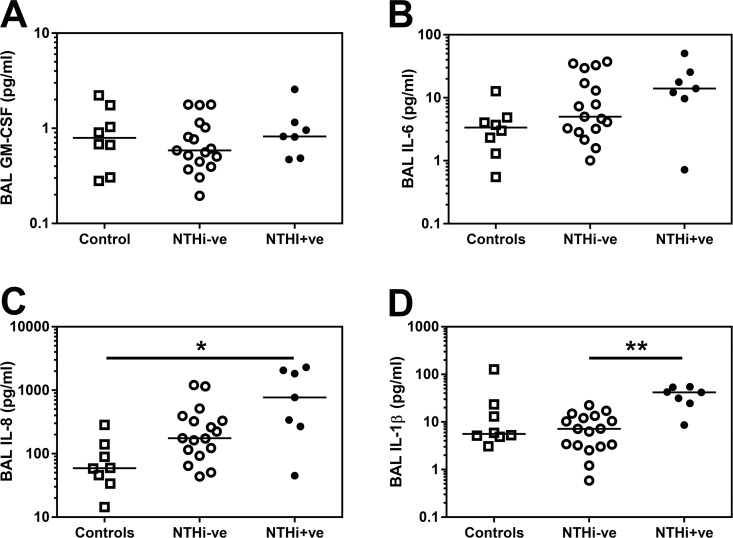
Inflammatory milieu is altered in NTHi+ve COPD BAL. **(A)** GM-CSF **(B)** IL-6, **(C)** IL-8 and **(D)** IL-1β in BAL derived from two lung lobes were analysed by Luminex multiplex assay and the average concentration from both lobes is presented. Open squares indicate controls, open circles indicate NTHi-ve COPD patients, closed circles indicate NTHi+ve patients. Bars represent median values and each dot represents an individual volunteer n = 8 for controls, n = 17 for NTHi-ve and n = 7 for NTHi+ve patients. Data were analysed using a Kruskal-Wallis ANOVA followed by a Dunn’s post hoc test. * p<0.05, ** p<0.01, *** p<0.01.

Of the eight cytokines measured, only GM-CSF, IL-1β, IL-6 and IL-8 were found in detectable amounts in BAL which is known to be a dilute compartment. We observed no differences in the amounts of GM-CSF or IL-6 (Fig 6A & 6B) between groups defined by NTHi infection. IL-8 was significantly increased in NTHi+ve patients compared to controls (Fig 6C), but IL-1β was significantly increased in BAL from the NTHi+ve airway compared to NTHI-ve patients (Fig 6D).

**Fig 7:**
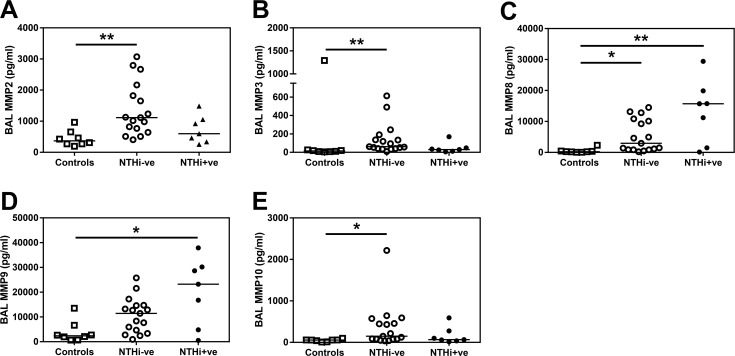
MMP levels are altered in COPD BAL. **(A)** MMP2, **(B)** MMP3, **(C)** MMP8, **(D)** MMP9 and **(E)** MMP10 in BAL derived from two lung lobes were analysed by Luminex multiplex assay and the average concentration from both lobes is presented. Open squares indicate controls, open circles indicate NTHi-ve COPD patients, closed circles indicate NTHi+ve patients. Bars represent median values and each dot represents an individual volunteer n = 8 for controls, n = 17 for NTHi-ve and n = 7 for NTHi+ve patients. Data were analysed using a Kruskal-Wallis ANOVA followed by a Dunn’s post hoc test. * p<0.05, ** p<0.01, *** p<0.01.

Detectable levels of all MMPs measured were found in COPD BAL and MMP2, 3, 8, 9 and 10 were significantly increased in BAL from COPD patients compared to controls (Fig 7A–7E). However, whilst there was a trend towards increased MMP8 and MMP9 in NTHI+ve BAL (Fig 7C & 7D) there was no significant difference in any MMP investigated between NTHi-ve and NTHi+ve COPD patients.

## Discussion

In the present study, we have demonstrated that there is an increased amount of total IgA, IgG and IgM in the COPD airway compared to controls. However, despite the presence of higher levels of functional and pathogen-specific antibody in the COPD airway, a significant proportion of our COPD cohort were infected with NTHi. Furthermore, these NTHI+ve patients had a significantly greater incidence of previous exacerbations than NTHi-ve volunteers in agreement with previous work which suggests that airway infection defines an important and longitudinally persistent endotype of COPD[2]. When we assessed the BAL obtained from patients we observed evidence of deficiency in both the amount and function of IgG1 in the NTHi+ve COPD airway. In contrast, IgA and IgM remained elevated in NTHi+ve BAL and there was also evidence of increased levels of IgM in the serum of NTHi+ve patients. In addition, there was evidence of systemic inflammation in the NTHi+ve cohort and the proinflammatory cytokine IL-1β was significantly elevated in the BAL of these patients.

An adaptive immune defect in COPD has been postulated for at least 40 years[10]. In that time there have been conflicting reports of changes in IgG, IgA and IgM in COPD that vary dependent on whether serum, sputum or lavage fluid was studied. For example, Groeneveld et al.[11] demonstrated *H*. *influenzae* colonisation of the airways despite the presence of specific IgA and IgG in the serum and sputum of COPD patients. More recently, Polosukhin[7] described a defect in sIgA distribution in BAL fluid from severe COPD patients, whilst in contrast IgA synthesis and accumulation was shown to be increased in COPD lung tissue[12]. Our data of increased IgA and sIgA in the COPD airway compared to health would support the observations of Groeneveld[11] and Ledjemi[12]. These observations may be a result of increased permeability of the mild-moderate COPD airway, rather than the impervious squamous metaplasic epithelium that has been demonstrated in more severe COPD[7], although we cannot rule out an increase in tissue resident B cells as previously described in COPD lung tissue[22].

However, differences in airway permeability are unlikely to account for the decrease in IgG1 we have observed in the NTHi-colonised airway, especially as we observe no serum defect in this isotype between NTHi-ve and NTHi+ve patients (Fig 4A). IgG1 is the most abundant human IgG isotype[23] and is thought to be largely involved in recognising protein antigens. As a non-encapsulated bacterium, NTHi immune correlates of protection from infection are likely to include IgG1 against Protein D, Protein F and outer membrane proteins, such as omp6, all previously demonstrated to be potential NTHi vaccine candidates[24–26]. Deficiencies in airway IgG1 may therefore predispose to infection of the COPD lung by this bacterium. Indeed, these observations may provide a rationale for the recent observation of the efficacy of intravenous immunoglobulin therapy in reducing the frequency of COPD exacerbations[27].

The reasons for this relative deficiency of IgG1 in the lung are open to speculation. As we have recently reported, there are a large number of proteases present in the COPD airway, notably the MMPs[28]. Several MMPs, as well as neutrophil elastase, are known to cleave IgG[29], and so we analysed the expression of these metalloproteases in our BAL samples. Whilst there was a trend for increased MMP8 and -9 in NTHi+ve BAL, we did not observe any statistically significant difference in MMP levels in BAL derived from NTHi+ve COPD patients compared to NTHI-ve patients. Thus, these data do not support a role for MMPs in degrading IgG1 in the NTHi+ve airway. Whilst we cannot rule out an effect of neutrophil elastase, there was no statistically significant difference in the proportion of neutrophils recovered in BAL between the two COPD groups (Table 1). NTHi expresses proteases, but only IgA is currently known to be targeted by the bacterial protease[30]. Among our NTHi isolates only one strain had the high expressing *igaB* allele and we observed no deficiency in IgA or sIgA in the NTHi+ve airway. Another reason for the observed decrease in IgG is suggested by a recent study demonstrating that inhaled steroids can inhibit both IgG gene expression and release[31]. However, we observed no difference in either the proportion of COPD patients taking inhaled steroids or the dose used between the infected and uninfected patients (Table 1). Therefore our data do not support a role for steroids in driving this IgG deficiency in these infected patients. Given the increase in IgM observed in the serum of these NTHi+ve patients, a further intriguing possibility is a failure of B cells to class switch from IgM to IgG in these patients.

IgM is produced by mature B cells in the secondary lymphoid organs upon antigen presentation and is usually of low affinity[32]. Once activated, B cells undergo class switch recombination (CSR) leading to production of higher affinity IgG isotypes. CSR is an intricate process involving signalling through the B cell receptor, CD40, Toll-like receptors and cytokine responses[32]. Smoking has previously been shown to affect B cell IgG switching in COPD[33], but we observed no significant difference in the levels of BAL IgG1 or serum IgM between COPD smokers (n = 11) and COPD ex-smokers (n = 13). IFNγ has been shown to suppress IgG1 production, whilst IL-6 can enhance production of all IgG isotypes[34]. Although we were unable to detect IFNγ in our BAL samples we have recently demonstrated an excess production of IFNy by COPD-derived T cells in an influenza infection model in the context of aberrant exhaustion signalling[9]. Hence excessive IFN production in the context of chronic infection may contribute to deficient isotype switching to IgG1. Although there was no difference in the levels of IL-6 between groups we did observe an increase in IL-1β in the BAL from NTHI+ve patients compared to NTHI-ve subjects, which may be a direct result of NTHi infection and stimulation of airway macrophages by the bacteria[35]. Interestingly, IL-1β has been implicated in the control of efficient IgM responses to influenza infection[36, 37] and gut IgA production[38]. Thus, it is possible that the increase in IL-1β in response to NTHi may support IgM and IgA production at the expense of decreased switching to IgG1. However as there was no correlation (p = 0.2938) between IL-1β and IgG1 release in the BAL of our COPD patients, further work is required to explore this conjecture.

We recognise that the main limitations of this study are the small sample size and that the data are associational rather than demonstrating a clear mechanism. However, despite the small numbers, we have demonstrated clear differences between health and COPD and between NTHi-infected and NTHi-ve COPD patients that are supported by data from previously published studies. Our COPD cohort was also restricted to mild and moderate patients due to the requirement for patients to undergo bronchoscopy. Changes in infection status and levels of Ig isotypes in BAL may indeed be more evident in more severe disease. A further limitation is that we have only used one strain of NTHi in our antibody binding assays. This was an attempt to limit assay variability that may be introduced due to different antigenic profiles amongst different bacterial strains. To increase the generalisability of our findings, future studies could consider using a pool of different strains.

In summary, overall the immunoglobulin isotypes tested were elevated in the airways of subjects with COPD, but there was a discrete deficiency in both the amount and function of IgG1 in BAL from COPD infected with NTHi. Whether this decrease in IgG1 is a cause or a consequence of infection by NTHi remains to be investigated but provides a potential therapeutic opportunity to develop new treatment modalities for this prevalent disease.

## Supporting Information

S1 Fig Increased NTHi-specific IgG but not IgG4 in COPD BAL. **(A)** Total IgG specific for the control strain of NTHi (3224A) in BAL were assessed by flow cytometry. Total IgG4 concentrations in **(B)** serum and **(C)** BAL derived from two lung lobes were analysed by MSD multiplex assay. Open squares indicate controls, open circles indicate NTHi-ve COPD patients, closed circles indicate NTHi+ve patients. Bars represent median values and each dot represents an individual volunteer n = 8 for controls, n = 17 for NTHi-ve and n = 7 for NTHi+ve patients. Data were analysed using a Kruskal-Wallis ANOVA followed by a Dunn’s post hoc test.
(PPTX)

S1 File **Table A, Characterisation of NTHi strains isolated from COPD airways.** NTHi strains isolated by culture from nasal brushes, sputum and bronchial brushes were whole genome sequenced and then MLST and *iga/igaB* alleles derived. Bronchial brushes were derived from different lung areas Right Lower Lobe (RLL), Right Upper Lobe (RUL) and Left Lower Lobe (LLL). **Table B, Clinical characteristics of COPD patients based on NTHi detection by culture alone.** Data are presented as median and IQR. Ex-smokers were defined as individuals who had stopped smoking for > 6 months. Median steroid dose is only calculated for volunteers taking steroids. Blood data shown represents 17 NTHI-ve and 5 NTHI+ve COPD volunteers. BAL data shown represents 15 NTHI-ve and 4 NTHI+ve COPD volunteers. # Two-tailed Mann Whitney U Test, † One-tailed Mann Whitney U test, ‡ Fishers Exact test.
(DOC)
