# Correction: Relationships between Mucosal Antibodies, Non-Typeable *Haemophilus influenzae* (NTHi) Infection and Airway Inflammation in COPD

**DOI:** 10.1371/journal.pone.0176749

**Published:** 2017-04-25

**Authors:** Karl J. Staples, Stephen Taylor, Steve Thomas, Stephanie Leung, Karen Cox, Thierry G. Pascal, Kristoffer Ostridge, Lindsay Welch, Andrew C. Tuck, Stuart C. Clarke, Andrew Gorringe, Tom M. A. Wilkinson

The images for Fig 2A and 2B are incorrectly identical. Please see the corrected Fig 2 here.

**Fig 2:**
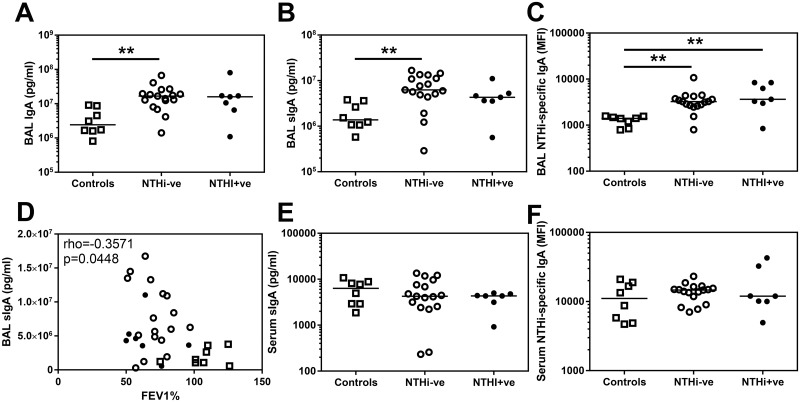
IgA and sIgA levels in BAL and serum derived from controls and COPD patients. (A) Total IgA and (B) secretory IgA in BAL derived from two lung lobes were analysed by MSD multiplex assay and ELISA respectively and the average concentration from both lobes is presented. (C) Assessment of BAL IgA specific for the control strain of NTHi (3224A) were assessed by flow cytometry. (D) Secretory IgA in BAL derived from all volunteers are plotted against FEV1% and analysed using a Spearman’s correlation with rho and p value presented. Assessment of (E) secretory IgA in serum measured by ELISA and (F) serum IgA specific for the control strain of NTHi (3224A) were assessed by flow cytometry. Open squares indicate controls, open circles indicate NTHi-ve COPD patients, closed circles indicate NTHi+ve COPD patients. Bars represent median values and each dot represents an individual volunteer n = 8 for controls and n = 17 for NTHi-ve COPD patients and n = 7 for NTHi+ve COPD patients. Data were analysed using a Kruskal-Wallis ANOVA followed by a Dunn’s post hoc test ** p<0.01.
