# Supplementary figures and images for: Relationships between Mucosal Antibodies, Non-Typeable Haemophilus influenzae (NTHi) Infection and Airway Inflammation in COPD
Source: PLoS One. 2016 Nov 29;11(11):e0167250. doi: 10.1371/journal.pone.0167250 (PMC5127575; doi:10.1371/journal.pone.0167250)

## Slide 1
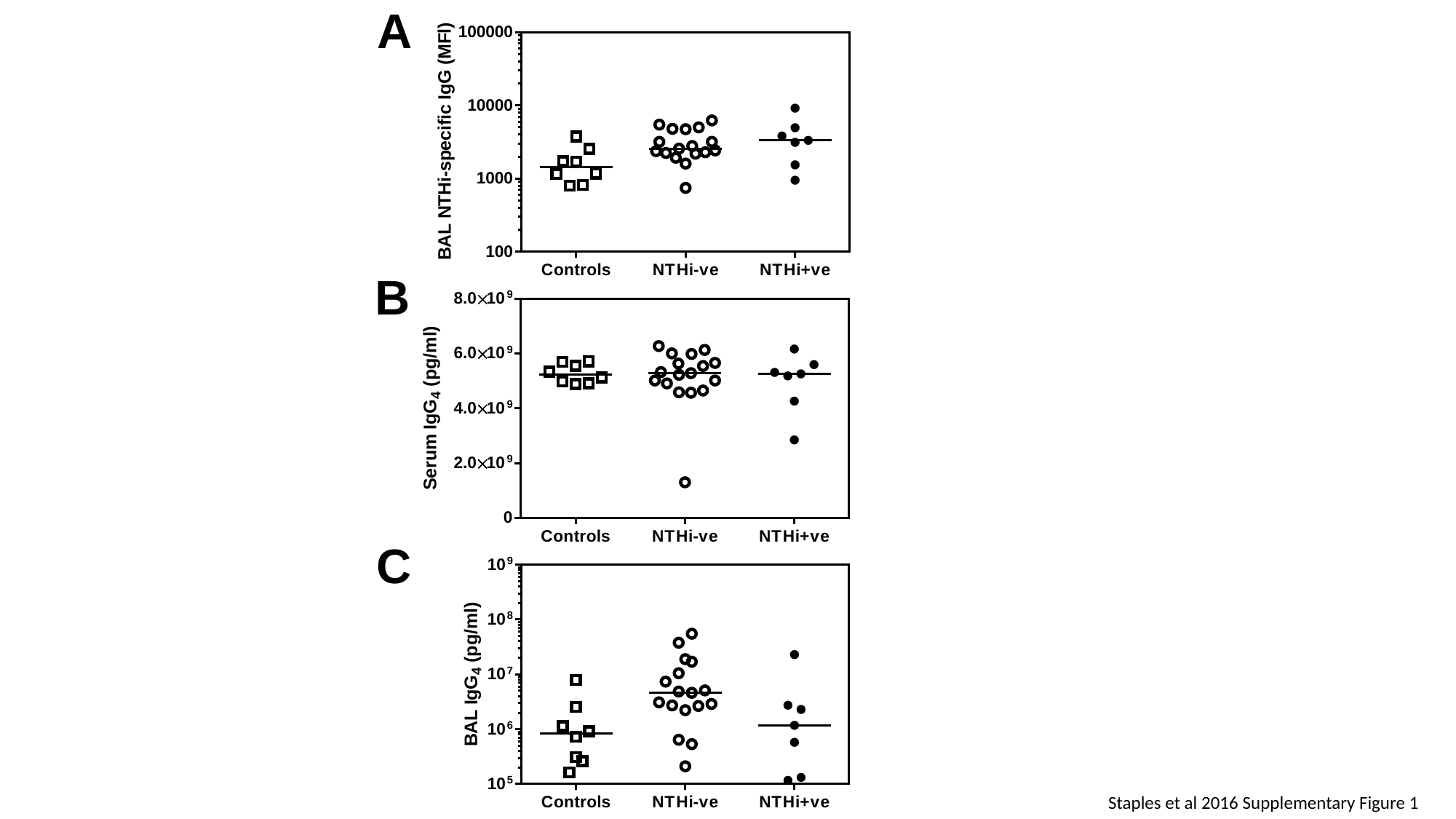

A
B
C
Staples et al 2016 Supplementary Figure 1

Supplement: S1 Fig — (A) Total IgG specific for the control strain of NTHi (3224A) in BAL were assessed by flow cytometry. Total IgG4 concentrations in (B) serum and (C) BAL derived from two lung lobes were analysed by MSD multiplex assay. Open squares indicate controls, open circles indicate NTHi-ve COPD patients, closed circles indicate NTHi+ve patients. Bars represent median values and each dot represents an individual volunteer n = 8 for controls, n = 17 for NTHi-ve and n = 7 for NTHi+ve patients. Data were analysed using a Kruskal-Wallis ANOVA followed by a Dunn’s post hoc test. (PPTX) [file pone.0167250.s001.pptx]
