# Supplementary material for: Relationships between Mucosal Antibodies, Non-Typeable Haemophilus influenzae (NTHi) Infection and Airway Inflammation in COPD
Source: PLoS One. 2016 Nov 29;11(11):e0167250. doi: 10.1371/journal.pone.0167250 (PMC5127575; doi:10.1371/journal.pone.0167250)
Supplement: S1 File — Table A, Characterisation of NTHi strains isolated from COPD airways. NTHi strains isolated by culture from nasal brushes, sputum and bronchial brushes were whole genome sequenced and then MLST and iga/igaB alleles derived. Bronchial brushes were derived from different lung areas Right Lower Lobe (RLL), Right Upper Lobe (RUL) and Left Lower Lobe (LLL). Table B, Clinical characteristics of COPD patients based on NTHi detection by culture alone. Data are presented as median and IQR. Ex-smokers were defined as individuals who had stopped smoking for > 6 months. Median steroid dose is only calculated for volunteers taking steroids. Blood data shown represents 17 NTHI-ve and 5 NTHI+ve COPD volunteers. BAL data shown represents 15 NTHI-ve and 4 NTHI+ve COPD volunteers. # Two-tailed Mann Whitney U Test, † One-tailed Mann Whitney U test, ‡ Fishers Exact test. (DOC) [file pone.0167250.s002.doc]

**Online supplement to :**

**Relationship of mucosal antibodies to non-typeable *Haemophilus influenzae* (NTHi) infection and airway inflammation in COPD**

Karl J. Staples^1,2*^, Stephen Taylor^3^, Steve Thomas^3^, Stephanie Leung^3^, Karen Cox^1^, Thierry G. Pascal^4^, Kristoffer Ostridge^5^, Lindsay Welch^5^, Andrew C. Tuck^1^, Stuart C. Clarke^1^, Andrew Gorringe^3^, Tom MA Wilkinson^1,2,5^.

**Results**

*NTHi strains isolated from COPD*

The MLST characteristics of NTHi strains isolated from COPD volunteers are presented in Supplementary Table A. Despite being isolated from different compartments, there were no differences in the strain of the bacteria.

IgA protease is an important virulence factor employed by *H. influenzae* to defend against the immunoglobulin A immune response by cleaving the antibody at the fab fragment . Two genes, *iga* and *igaB* can encode it and *iga* has been found in all *H. influenzae* whereas *igaB* has only been identified in approximately a third . In contrast to *igaB*, a considerable level of sequence variation has been shown for *iga* . Invasive strains have displayed higher levels of IgA protease as have *igaB* positive *H. influenzae* . In addition *igaB* positive strains have been discovered more prevalently in those isolated from COPD rather than otitis media or nasopharyngeal carriage .

Whilst all of the isolated strains were *iga* positive, only one strain (ST57) was *igaB* positive (Supplementary Table A).

|  | ST | adk | atpG | frdB | fucK | mdh | pgi | recA | iga | igaB |
| --- | --- | --- | --- | --- | --- | --- | --- | --- | --- | --- |
| MICA-14 RLL brush | 57 | 14 | 7 | 13 | 7 | 17 | 13 | 17 | + | + |
| MICA-14 RUL brush | 57 | 14 | 7 | 13 | 7 | 17 | 13 | 17 | + | + |
| MICA-14 sputum | 57 | 14 | 7 | 13 | 7 | 17 | 13 | 17 | + | + |
| MICA-18 nasal brush | 103 | 1 | 1 | 1 | 14 | 9 | 14 | 13 | + | - |
| MICA-18 RUL | 103 | 1 | 1 | 1 | 14 | 9 | 14 | 13 | + | - |
| MICA-18 sputum | 103 | 1 | 1 | 1 | 14 | 9 | 14 | 13 | + | - |
| MICA-20 LLL brush | 1447 | 18 | 6 | 19 | 7 | 10 | 159 | 12 | + | - |
| MICA-20 RUL brush | 1447 | 18 | 6 | 19 | 7 | 10 | 159 | 12 | + | - |
| MICA-20 sputum | 1447 | 18 | 6 | 19 | 7 | 10 | 159 | 12 | + | - |
| MICA-29 nasal brush | 408 | 1 | 1 | 1 | 1 | 1 | 21 | 5 | + | - |
| MICA-29 RLL brush | 408 | 1 | 1 | 1 | 1 | 1 | 21 | 5 | + | - |
| MICA-29 RUL brush | 408 | 1 | 1 | 1 | 1 | 1 | 21 | 5 | + | - |
| MICA-29 sputum | 408 | 1 | 1 | 1 | 1 | 1 | 21 | 5 | + | - |
| MICA-33 RLL brush | 14 | 5 | 1 | 1 | 1 | 1 | 2 | 5 | + | - |

**Table A:** **Characterisation of NTHi strains isolated from COPD airways.** NTHi strains isolated by culture from nasal brushes, sputum and bronchial brushes were whole genome sequenced and then MLST and *iga*/*iga*B alleles derived. Bronchial brushes were derived from different lung areas Right Lower Lobe (RLL), Right Upper Lobe (RUL) and Left Lower Lobe (LLL).

|  | NTHi-ve | NTHI+ve | p Value |
| --- | --- | --- | --- |
| N | 19 | 5 | - |
| Age (years) | 66  (56.00 – 70.00) | 68  (61.00 – 71.00) | 0.5457# |
| Gender, M/F | 14 / 5 | 2 / 3 | 0.2885‡ |
| BMI | 29.03  (26.59 – 32.48) | 28.13  (26.50 – 30.04) | 0.6382# |
| Current Smoker | 8 | 3 | 0.6299‡ |
| Pack Years | 38.00  (30.00 – 60.00) | 50.00  (11.00 – 85.00) | >0.9999# |
| FEV_1_% | 70.00  (57.00 – 80.00) | 64.00  (57.00 – 86.00) | 0.9309# |
| FEV_1_/FVC ratio | 0.56  (0.51 – 0.62) | 0.53  (0.46 – 0.62) | 0.5891# |
| Exacerbations in previous year, N | 1.0  (1.0 – 2.0) | 3.0  (3.0 – 6.5) | **0.0017†** |
| Blood WCC, 10^9^/L | 6.60  (5.50 – 7.60) | 8.30  (6.90 – 10.45) | 0.0583# |
| Blood neutrophils, 10^9^/L | 3.70  (3.10 – 4.45) | 5.20  (4.05 – 6.55) | **0.0327#** |
| Blood lymphocytes, 10^9^/L | 2.00  (1.85 – 2.40) | 2.60  (2.05 – 3.00) | 0.0819# |
| BAL macrophage, % | 76.00  (46.50 – 89.50) | 91.50  (72.75 – 93.75) | 0.0859# |
| BAL neutrophil, % | 5.00  (2.00 – 8.00) | 2.50  (2.00 – 22.50) | 0.9396# |
| BAL total IgG1 (µg/ml) | 72.18  (48.81 – 83.45) | 33.94  (33.05 – 48.52) | **0.0053#** |
| BAL NTHi-specific IgG1 (MFI) | 101.9  (63.75 – 132.60) | 38.85  (-10.05 – 116.60) | 0.0602† |
| BAL total IgA (µg/ml) | 16.93  (12.48 – 19.52) | 10.58  (3.82 – 48.23) | 0.3306# |
| BAL total IgM (µg/ml) | 1.66  (0.97 – 2.83) | 1.12  (0.57 – 6.40) | 0.8365# |
| BAL IL-1β (pg/ml) | 7.20  (3.24 – 13.41) | 41.79  (28.15 – 47.92) | **0.0009#** |

**Table B: Clinical characteristics of COPD patients based on NTHi detection by culture alone.** Data are presented as median and IQR. Ex-smokers were defined as individuals who had stopped smoking for > 6 months. Median steroid dose is only calculated for volunteers taking steroids. Blood data shown represents 17 NTHI-ve and 5 NTHI+ve COPD volunteers. BAL data shown represents 15 NTHI-ve and 4 NTHI+ve COPD volunteers. # Two-tailed Mann Whitney U Test, † One-tailed Mann Whitney U test, ‡ Fishers Exact test.

**References**

1. Vitovski S, Dunkin KT, Howard AJ, Sayers JR. Nontypeable Haemophilus influenzae in carriage and disease: a difference in IgA1 protease activity levels. JAMA. 2002;287(13):1699-705. PubMed PMID: 11926894.

2. Fernaays MM, Lesse AJ, Cai X, Murphy TF. Characterization of igaB, a second immunoglobulin A1 protease gene in nontypeable Haemophilus influenzae. Infection and immunity. 2006;74(10):5860-70. doi: 10.1128/IAI.00796-06. PubMed PMID: 16988265; PubMed Central PMCID: PMCPMC1594874.

3. McCrea KW, Xie J, LaCross N, Patel M, Mukundan D, Murphy TF, et al. Relationships of nontypeable Haemophilus influenzae strains to hemolytic and nonhemolytic Haemophilus haemolyticus strains. J Clin Microbiol. 2008;46(2):406-16. doi: 10.1128/JCM.01832-07. PubMed PMID: 18039799; PubMed Central PMCID: PMCPMC2238123.

4. Murphy TF, Lesse AJ, Kirkham C, Zhong H, Sethi S, Munson RS, Jr. A clonal group of nontypeable Haemophilus influenzae with two IgA proteases is adapted to infection in chronic obstructive pulmonary disease. PloS one. 2011;6(10):e25923. doi: 10.1371/journal.pone.0025923. PubMed PMID: 21998721; PubMed Central PMCID: PMCPMC3187821.

5. Poulsen K, Reinholdt J, Kilian M. A comparative genetic study of serologically distinct Haemophilus influenzae type 1 immunoglobulin A1 proteases. J Bacteriol. 1992;174(9):2913-21. PubMed PMID: 1373717; PubMed Central PMCID: PMCPMC205944.

6. Kilian M, Poulsen K. Enzymatic, serologic, and genetic polymorphism of Haemophilus influenzae IgA1 proteases. The Journal of infectious diseases. 1992;165 Suppl 1:S192-3. PubMed PMID: 1588161.
